# Rodents as intermediate hosts of cestode parasites of mammalian carnivores and birds of prey in Poland, with the first data on the life-cycle of *Mesocestoides melesi*

**DOI:** 10.1186/s13071-020-3961-2

**Published:** 2020-02-22

**Authors:** Anna Bajer, Mohammed Alsarraf, Dorota Dwużnik, Ewa J. Mierzejewska, Marta Kołodziej-Sobocińska, Jolanta Behnke-Borowczyk, Łukasz Banasiak, Maciej Grzybek, Katarzyna Tołkacz, Natalia Kartawik, Łukasz Stańczak, Patrycja Opalińska, Małgorzata Krokowska-Paluszak, Grzegorz Górecki, Mustafa Alsarraf, Jerzy M. Behnke

**Affiliations:** 1 Department of Eco-Epidemiology of Parasitic Diseases, Faculty of Biology, University of Warsaw, Miecznikowa 1, 02-096 Warsaw, Poland; 2 Mammal Research Institute, Polish Academy of Sciences, Stoczek 1c, 17-230 Białowieża, Poland; 3 Department of Forest Phytopathology, Faculty of Forestry, Poznań University of Life Sciences, Poznan, Poland; 4 Department of Molecular Phylogenetics and Evolution, Institute of Botany, Faculty of Biology, University of Warsaw, Biological and Chemical Research Centre, Żwirki i Wigury 101, 02-089 Warsaw, Poland; 5 Department of Tropical Parasitology, Institute of Maritime and Tropical Medicine, University of Gdansk, Powstania Styczniowego 9B, 81-519 Gdynia, Poland; 6 Department of Game Management and Forest Protection, Faculty of Forestry, Poznań University of Life Sciences, Poznan, Poland; 7 School of Life Sciences, University of Nottingham, University Park, Nottingham, NG7 2RD UK

**Keywords:** *Mesocestoides*, *Hydatigera*, *Taenia crassiceps*, Rodents, Fox, Badger, Lynx

## Abstract

**Background:**

Rodents constitute an important part of the diet of many carnivore species. This predator-prey food chain is exploited by helminth parasites, such as cestodes, whose larval stages develop in rodents and then mature to the adult stage in predators. The main aim of our study was to use molecular techniques for identification of cestode species recovered from both intermediate and definitive hosts, with a particular focus on the genus *Mesocestoides.*

**Methods:**

Larval cestodes were obtained during our long-term studies on rodent helminth communities in the Mazury Lake District in the north-east Poland in 2000–2018. Cestode larvae/cysts were collected from body cavities or internal organs (e.g. liver) during autopsies. Adult tapeworms were derived from nine red foxes, three Eurasian badgers and one Eurasian lynx. PCR amplification, sequencing and phylogenetic analyses were conducted employing three genetic markers: *18S* rDNA, mitochondrial (mt) *12S* rDNA and the mt cytochrome *c* oxydase subunit 1 (*cox*1) gene fragment.

**Results:**

Altogether 19 *Mesocestoides* samples were analyzed, including 13 adult tapeworms from definitive hosts and six larval samples from 4 bank voles and 2 yellow-necked mice. Phylogenetic analyses revealed three well-supported trees of similar topology. In each case the *Mesocestoides* samples formed two separate clades. All isolates from foxes, the lynx isolate and two isolates from rodents grouped with *Mesocestoides litteratus*. Four isolates from rodents and all three isolates from Eurasian badgers were resolved in a separate clade, most similar to North American *M. vogae* (syn. *M. corti*). Examination of fixed, stained adult specimens from Eurasian badgers revealed consistency with the morphology of *Mesocestoides melesi*. Therefore, this clade is likely to represent *M. melesi*, a species first described in 1985 from the Eurasian badger *Meles meles*. Molecular analysis allowed also the identification of *Taenia crassiceps*, *Hydatigera kamiyai* and *Cladotaenia globifera* among larvae derived from rodents.

**Conclusions:**

Molecular and phylogenetic analyses support the recognition of *M. melesi* as a valid species. Our data represent the first record of the larvae of this species in rodents. This is the first report on the occurrence of *H. kamiyai* in rodents from Poland.
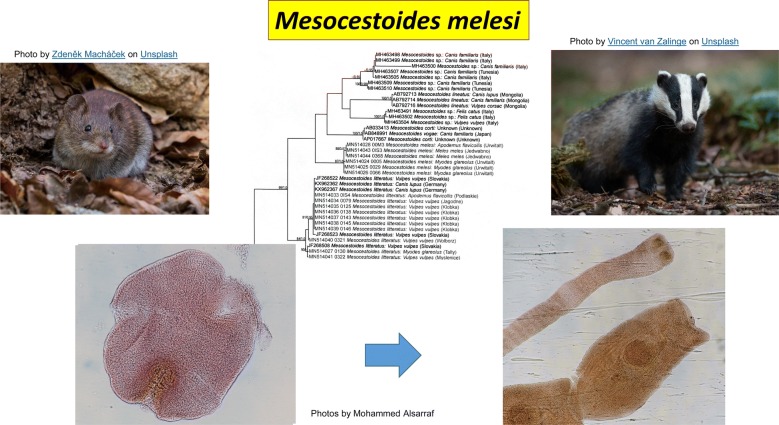

## Background

Rodents constitute an important part of the diet of many carnivorous species. This predator-prey food chain is exploited by helminth parasites, such as cestodes, whose larval stages develop in rodents and then mature to the adult stage in predators (both carnivorous mammals and birds of prey). The role of rodents as obligatory intermediate or paratenic hosts of tapeworms exploiting this route of transmission (families Mesocestoididae, Taeniidae and Paruterinidae) is therefore indispensable in enabling the completion of their life-cycles.

In our previous studies on parasite communities of rodents from north-east Poland, we investigated the larval cestodes present in different body cavities and in the liver [1–5]. The larval stages of several cestode species were recognized in bank voles (*Myodes glareolus)* by morphological features, including *Mesocestoides* sp., *Cladotaenia globifera*, *Taenia martis*, *Taenia mustelae* and *Hydatigera taeniaeformis* (syn. *Taenia taeniaeformis*). However, in recent years molecular studies have revealed that some of these species actually comprise complexes that include cryptic species which could not be distinguished earlier by conventional morphological examination. Hence re-description of these species has been necessary and driven primarily by their genetic signatures, i.e. *H. taeniaeformis* parasitizing voles has been re-described as *Hydatigera kamiyai* and *T. mustelae* as *Versteria mustelae* [6, 7]. To the best of our knowledge, no such molecular studies, reporting the presence of newly raised species, have been carried out to date on cestode isolates from rodents in Poland.

Tapeworms of the genus *Mesocestoides* (Cyclophyllidea, Mesocestoididae) have been reported to parasitize a range of wild and domestic carnivores and even birds of prey as definitive hosts [8–10]. The systematics of *Mesocestoides* spp. is still not fully resolved [11, 12] and the unarmed scolex and pleomorphic metacestodes/larvae (tetrathyridia) found in rodents and other intermediate hosts (insectivore mammals, birds, reptiles, etc.), do not provide sufficient characteristic features to enable unambiguous differentiation between species. To date, 4–7 *Mesocestoides* species have been reported from Europe [13–17]. The two most commonly reported species are *M. litteratus* found in red foxes (originally described as from a ‘fox’), rodents, grey wolves, dogs and cats among others; and *M. lineatus* that has been reported from domestic/wild cats (originally described from wild cats, *Felis sylvestris*) and dogs, jackals and other carnivores [18]. In Poland, only one molecular study has been completed on *Mesocestoides* larvae from rodent hosts, and this identified *M. litteratus* in striped field mice *Apodemus agrarius* and *M. glareolus* from the Wrocław area (western Poland) [19]. Red foxes (*Vulpes vulpes*) are considered to be the principal hosts of adult *Mesocestoides* spp. in Poland [20]. In recent years we have carried out extensive studies on different parasites of the red fox from different regions of Poland and we have confirmed the high overall prevalence of *Mesocestoides* in foxes, with a prevalence of 88% in all of the sampled populations [21], as in Karamon et al. [20].

The main aim of our current study was to use molecular techniques for identification of, and comparison between, cestode species recovered from both intermediate and definitive hosts: sylvatic rodents, red foxes and other definitive hosts, with a particular focus on *Mesocestoides* spp.

## Methods

Larval cestodes were obtained during our long-term studies on rodent helminths in the Mazury Lake District in north-east Poland in 2000–2018 [1–5]. In addition, one *Mesocestoides* sample was obtained from a yellow-necked mouse (*Apodemus flavicollis*) from the Białowieża Forest region, north-east Poland. Altogether, ten infected rodents were examined, including five bank voles *M. glareolus*, two yellow-necked mice *A. flavicollis*, two common voles *Microtus arvalis* and one striped field mouse *A. agrarius* (Table 1). Cestode larvae from body cavities, identified preliminarily as *Mesocestoides* spp., were obtained from seven rodents, including one sample identified later by molecular typing as an undeveloped *Hydatigera* larva. In one sample, cysts found in the body cavity were morphologically identified as *T. crassiceps*. Two larval samples were derived from rodent livers: one mature strobilocercus of *Hydatigera* sp. and numerous *C. globifera* larvae. The host species for each specimen are recorded in Table 1.

**Table 1:** Origin (host species, region and site) and results of genotyping for larval and adult cestodes involved in the study

Host group	Host ID	Host species	Region, site, year	Developmental stage, localization	Cestode species (morphological)	Cestode species (molecular)	GenBank ID		
							*18S* rDNA	*12S* rDNA	*cox*1
Rodents	0005	*M. glareolus*	Masuria, U, 2018	Larvae, PC	*Mesocestoides* sp.	*M. melesi*	MN512706	MN505192	MN514024
	0029	*M. glareolus*	Masuria, U, 2018	Larvae, PC	*Mesocestoides* sp.	*M. melesi*	MN512707	MN505193	MN514025
	0066	*M. glareolus*	Masuria, U, 2018	Larvae, PC	*Mesocestoides* sp.	*M. melesi*	MN401347	MN505194	MN514026
	0130	*M. glareolus*	Masuria, T, 2018	Larvae, PC, Liv	*Mesocestoides* sp.	*M. litteratus*	MN401340	MN505195	MN514027
	00M3	*A. flavicollis*	Masuria, U, 2001	Larvae, PC	*Mesocestoides* sp.	*M. melesi*	MN401345	MN505196	MN514028
	0177	*A. agrarius*	Masuria, T, 2018	Larvae, Liv	*C. globifera*	*C. globifera*	nd	MN505197	MN514029
	0D45	*M. arvalis*	Masuria, U, 2000	Larvae, Liv	*H. taeniaeformis*	*H. kamiyai*	nd	MN505198	MN514030
	0D53	*M. arvalis*	Masuria, U, 2000	Larvae, PC	*T. crassiceps*	*T. crassiceps*	nd	nd	MN514031
	D172	*M. glareolus*	Masuria, U, 2000	Larvae, PC	*Mesocestoides* sp.	*H. kamiyai*	nd	nd	MN514032
	0IS4	*A. flavicollis*	Podlaskie, B, 2016	Larvae, PC	*Mesocestoides* sp.	*M. litteratus*	MN401344	MN505199	MN514033
Canids	0079	*V. vulpes*	Mazovia, J, 2017	Adult, SI	*M. litteratus*	*M. litteratus*	MN401342	MN505200	MN514034
	0125	*V. vulpes*	Kujawsko-Pomorskie, K, 2017	Adult, SI	*M. litteratus*	*M. litteratus*	MN512708	MN505201	MN514035
	0138	*V. vulpes*	Kujawsko-Pomorskie, K, 2017	Adult, SI	*M. litteratus*	*M. litteratus*	MN512709	nd	MN514036
	0143	*V. vulpes*	Kujawsko-Pomorskie, K, 2017	Adult, SI	*M. litteratus*	*M. litteratus*	MN401343	MN505202	MN514037
	0145	*V. vulpes*	Kujawsko-Pomorskie, K, 2017	Adult, SI	*M. litteratus*	*M. litteratus*	MN512710	nd	MN514038
	0146	*V. vulpes*	Kujawsko-Pomorskie, K, 2017	Adult, SI	*M. litteratus*	*M. litteratus*	MN512711	MN505203	MN514039
	0321	*V. vulpes*	Łódzkie, Wo, 2018	Adult, SI	*M. litteratus*	*M. litteratus*	MN401341	MN505204	MN514040
	0322	*V. vulpes*	Łódzkie, M, 2018	Adult, SI	*M. litteratus*	*M. litteratus*	MN512712	MN505205	MN514041
	0280	*V. vulpes*	Mazovia, W, 2018	Adult, SI	*T. crassiceps*	*T. crassiceps*	MN512713	MN505206	MN514042
Mustelids	0366	*M. meles*	Masuria, Je, 2018	Adult, SI	*Mesocestoides* sp.	*M. melesi*	MN512714	MN505207	nd
	0367	*M. meles*	Masuria, Je, 2018	Adult, SI	*Mesocestoides* sp.	*M. melesi*	MN401346	MN505208	MN514043
	0368	*M. meles*	Masuria, Je, 2018	Adult, SI	*Mesocestoides* sp.	*M. melesi*	nd	MN505209	MN514044
Felids	0IS1	*L. lynx*	Podkarpackie, L, 2013	Adult, SI	*Mesocestoides* sp.	*M. litteratus*	nd	MN505210	nd

*Abbreviations*: U, Urwitałt; T, Tałty; W, Warsaw; J, Jagodne; K, Kłóbka; Wo, Wolbórz, My, Myślenice, Je, Jedwabno, B, Białowieża, L, Lubaczów; PC, peritoneal cavity; Liv, liver; SI, small intestine; nd, not done

Adult *Mesocestoides* tapeworms were selected from eight red foxes (*V. vulpes*) originating from three administrative regions of Poland: the Mazowieckie, Łódzkie and Kujawsko-Pomorskie Voivodeships (Table 1). One adult *T. crassiceps* from a red fox was also included in the study for comparison with rodent samples. Additionally, adult *Mesocestoides* specimens from one Eurasian lynx (*Lynx lynx*) [22] and from three Eurasian badgers (*Meles meles*), from Podkarpackie Voivodeship, south-east Poland and the Mazury Lake District, north-east Poland, respectively, were also included (Table 1).

### Morphological examination of *Mesocestoides* spp.

Larval *Mesocestoides* from rodents and adult *Mesocestoides* from badgers were flattened and fixed in AFA solution (100 ml 40% formaldehyde, 250 ml 95% ethanol, 100 ml glycerine, 50 ml glacial acetic acid, 500 ml distilled water) and stained using borax carmine, dehydrated in an ethanol series and mounted in Canada balsam for microscopical examination. Slides were examined and selected measurements were recorded using a NIKON Eclipse E-600 microscope with differential interference contrast, equipped with the NIS Elements Br 3.1 software (Nikon Instruments Co., Tokyo, Japan) for image processing and recording. Photographs were taken using a NIKON DX-1200 digital camera connected to the microscope.

### DNA extraction and amplification

Genomic DNA was extracted from specimens fixed in ethanol (about 20 mg of tissue) using the DNAeasy Blood & Tissue kit (Qiagen, Hilden, Germany) and stored at a temperature of − 20 °C.

Molecular typing of tapeworms was performed by amplification and sequencing of three markers: (i) a fragment of *c.*1100 bp of *18S* rDNA was amplified using the primers Worm A (5′-GCG AAT GGC TCA TTA AAT AG-3′) and 1270R (5′-CCG TCA ATT CCT TTA AGT TT-3′) [23]; (ii) a fragment of *c.*350 bp of mitochondrial (mt) *12S* rDNA was amplified using the primers P60 for (5′-TTA AGA TAT ATG TGG TAC AGG ATT AGA TAC CC-3′) and P375 rev (5′-AAC CGA GGG TGA CGG GCG GTG TGT ACC-3′) [24]; (iii) a fragment of *c.*400 bp of the cytochrome *c* oxidase subunit 1 (*cox*1) was amplified using the primers JB3 (5′-TTT TTT GGG CAT CCT GAG GTT TAT-3′) and JB45 (5′-TAA AGA AAG AAC ATA ATG AAA ATG-3′) [25]. The PCR reactions were performed in a volume of 20 μl, including 1× PCR Dream Taq Green buffer (Thermo Fisher Scientific, Waltham, Massachusetts, USA), 1U Dream Taq polymerase (Thermo Fisher Scientific), 0.33 mM dNTPs, 1 μM of each primer and 2 μl of the extracted DNA sample. Negative controls were performed with nuclease-free distilled water, in the absence of template DNA.

All PCR reactions were carried out in identical cycling conditions: primary denaturation at 94 °C for 3 min, followed by 40 cycles of denaturation at 94 °C for 30 s, annealing at 56 °C for 1 min, and elongation at 72 °C for 1 min, followed by a final elongation step at 72 °C for 7 min and a hold step at 4 °C.

PCR products were subjected to electrophoresis on a 1.5% agarose gel, stained with Midori Green stain (Nippon Genetics, GmbH). PCR products were directly sequenced in both directions by Genomed S.A. (Warsaw, Poland) with the primers used for DNA amplification. Sequences were aligned and visually inspected using Clustal W in MEGA v.7.0 [25]. Consensus sequences were compared with sequences deposited in the GenBank database.

Phylogenetic analyses were conducted separately for each molecular marker (Table 2). Sequences were aligned using E-INS-i algorithm implemented in Mafft version 7.271 [26, 27]. Maximum likelihood trees were obtained in RAxML version 8.2.4 [28] assuming a GTR + G model for the nucleotide substitution process. The topology and branch lengths were optimized starting the analysis 200 times with distinct randomized maximum parsimony trees. Branch support values were obtained during 1000 rapid bootstrap replicates. Bayesian phylogenetic inference was conducted in MrBayes parallel version 3.2.6 [29] with selection of the model of nucleotide substitution (for *12S* rDNA: GTR + G; for *18S* rDNA: K80 + G; for *cox*1: GTR + G) by using the BIC implemented in Partition Finder2 [30, 31]. The Bayesian analysis was run for 10 million generations with two independent runs sampled every 1000 generations. The results were combined after discarding 25% of trees considered as ‘burn-in’ phase. The remaining 30,000 trees were summarized as a 50% majority rule consensus tree. Convergence of independent runs and the effective sample size of sampled parameters were inspected in Tracer version 1.6.

**Table 2:** Characteristics of the nucleotide datasets used in phylogenetic analyses

	*12S* rDNA	*18S* rDNA	*cox*1
Number of sequences	51	33	63
Sequence length variation (bp)	222–337	593–1133	366–388
Number of aligned positions			
Total	350	1206	388
Constant	193	1010	235
Autapomorphic	28	86	9
Parsimony informative	129	110	144
Containing gaps	171	707	29
Percentage of gaps/missing data	10.35	11.42	0.93

## Results

### Molecular identification of *Mesocestoides* spp.

**Fig. 1:**
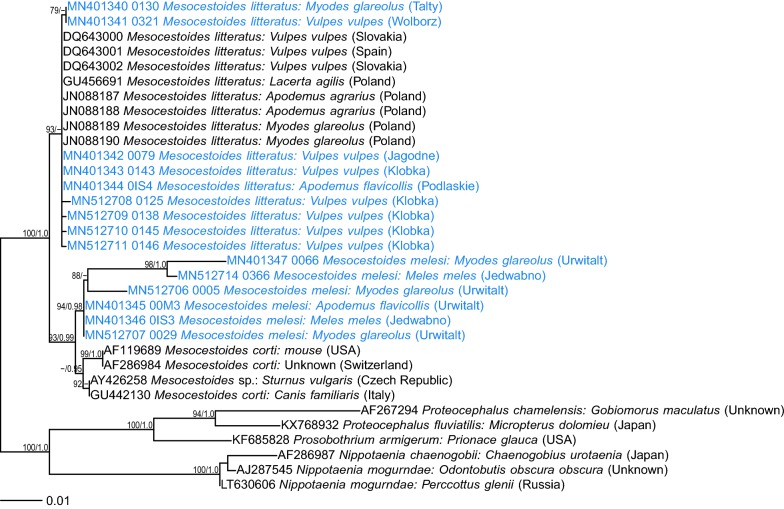
Maximum likelihood tree for *Mesocestoides* and relatives based on *18S* rDNA (GTR + G model). Numbers along branches are bootstrap support (BS) and posterior probability (PP) values if corresponding bipartition was found in Bayesian 50% majority rule consensus tree. Only values of BS higher than 75% and PP higher than 0.95 are shown. The scale-bar indicates the expected number of nucleotide substitutions per site

**Fig. 2:**
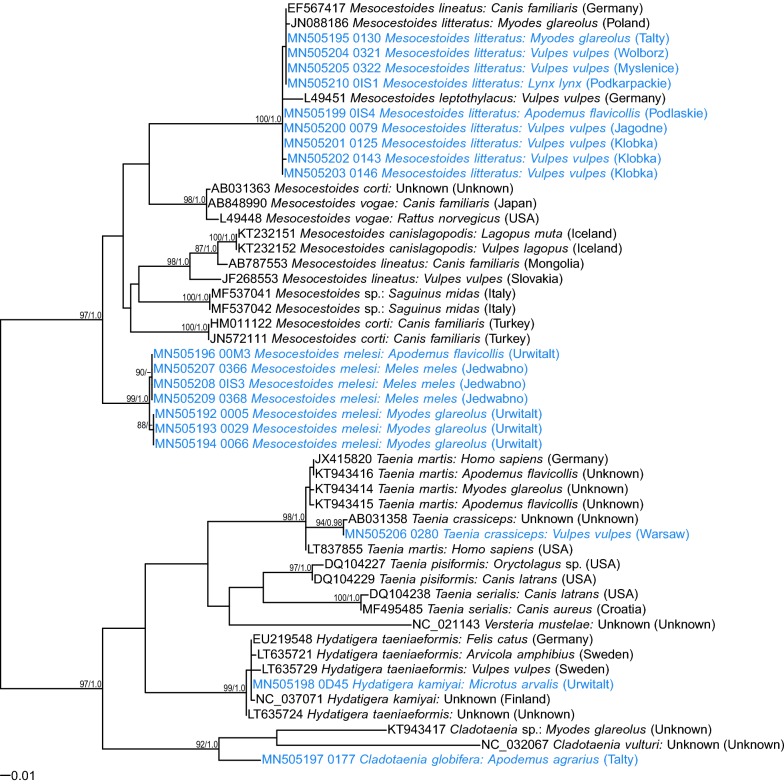
Maximum likelihood tree for *Mesocestoides* and relatives based on mt *12S* rDNA (GTR + G model). Numbers along branches are bootstrap support (BS) and posterior probability (PP) values if corresponding bipartition was found in Bayesian 50% majority rule consensus tree. Only values of BS higher than 75% and PP higher than 0.95 are shown. The scale-bar indicates the expected number of nucleotide substitutions per site

**Fig. 3:**
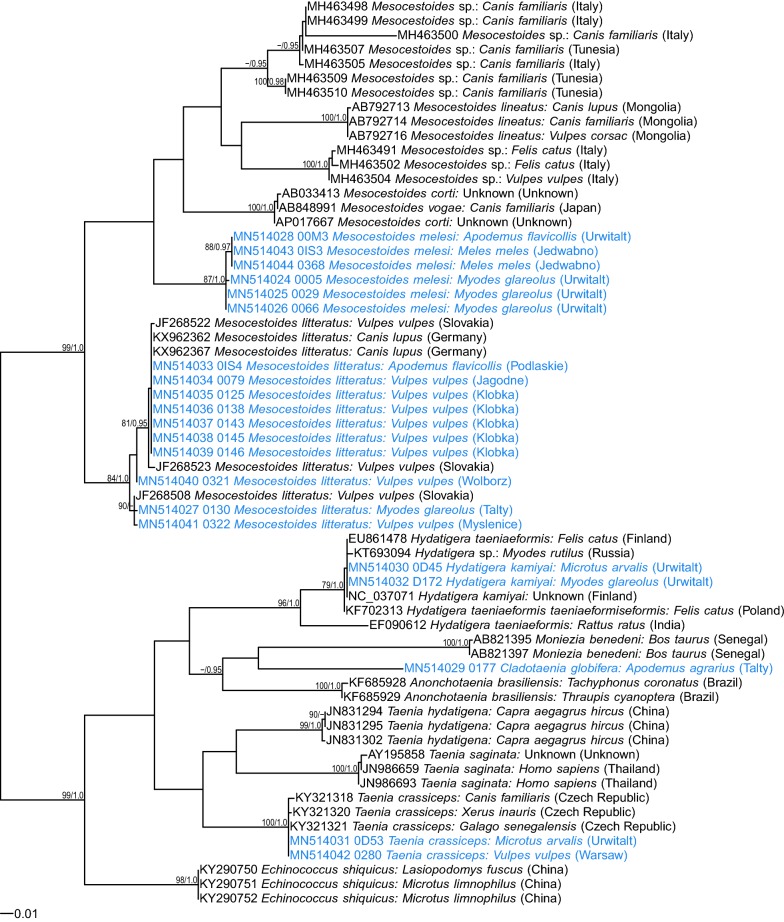
Maximum likelihood tree for *Mesocestoides* and relatives based on *cox*1 gene fragment (GTR + G model). Numbers along branches are bootstrap support (BS) and posterior probability (PP) values if corresponding bipartition was found in Bayesian 50% majority rule consensus tree. Only values of BS higher than 75% and PP higher than 0.95 are shown. The scale-bar indicates the expected number of nucleotide substitutions per site

All eight adult *Mesocestoides* specimens from red foxes and one adult *Mesocestoides* from the Eurasian lynx were identified as *M. litteratus* based on 98–100% identity of the three markers with *M. litteratus* sequences deposited in GenBank (Additional file 1: Tables S1–S3). All three applied genetic markers were successful in amplifying *Mesocestoides* spp. DNA from foxes; however, only *12S* rDNA could be amplified from the lynx sample. All the sequences obtained in the present study grouped with sequences of *M. litteratus* from carnivores from a range of European countries (Figs. 1, 2, 3).

Among six larval *Mesocestoides* isolates from rodents, only two (one from a bank vole from Masuria and one from a yellow-necked mouse from Białowieża) were identified as *M. litteratus*, based on 98–100% identity of the three markers used for analysis with *M. litteratus* sequences deposited in GenBank (Additional file 1: Tables S1–S3). A group of four sequences could not be identified due to the lack of identical sequences of *18S* rDNA, mt *12S* rDNA and *cox*1 in the GenBank database. These sequences, one derived from *A. flavicollis* and three from *M. glareolus*, both from the Mazury Lake District, displayed the highest similarity (97.4–99.4%) to *M. vogae* (syn. *M. corti*) based on *18S* rDNA (Additional file 1: Table S2). Based on mt *12S* rDNA and *cox*1 sequences, percent similarity was markedly lower (90.2–90.5% in *12S* rDNA and 88–89% in *cox*1; Additional file 1: Tables S1, S3), suggesting the presence of a distinct species.

In phylogenetic analyses, these four isolates grouped separately (Figs. 1, 2, 3), distant from *M. litteratus*, *M. lineatus* or *M. canislagopodis*, but displaying closer similarity with North American *M. vogae* (syn. *M. corti*) (Figs.1, 3). Maximum likelihood and Bayesian trees had very similar topology and therefore we show only ML trees with posterior probability for corresponding bipartitions (Figs. 1, 2, 3).

Interestingly, all three *Mesocestoides* sequences derived from adult worms from Eurasian badgers were very similar (Additional file 1: Tables S1–S3) to these four isolates from rodents. In all phylogenetic trees, the four sequences from rodents and all available sequences from badgers formed one phylogenetic group, distant from *M. litteratus*, other species and a range of recently identified *Mesocestoides* genotypes from Italy and Tunisia [32, 33]. This group of sequences displayed the highest similarity to *M. vogae* (syn. *M. corti*) based on *18S* rDNA and *cox*1 markers (Figs. 1, 3). Some minor diversity (1–3 SNPs) among this group of sequences was also observed (Figs. 1, 2, 3; Additional file 1: Tables S1–S3). There were also some differences between different *M. litteratus* sequences/isolates (Figs. 2, 3; *12S* and *cox*1).

### Morphological examination of *Mesocestoides* spp.

The larvae of putative *M. melesi* were half the size of *M. litteratus* larvae (Additional file 2: Figure S1) and additional morphological evaluation of slides with stained adult tapeworms from Eurasian badgers (Additional file 3: Figure S2) revealed no obvious differences between the present cestodes and these described as *M. melesi*. Although mean sucker length and width of the adult tapeworms from badgers were slightly larger than the means reported by Yanchev and Petrov [34] (Additional file 4: Table S4), they were well within the range described for *M. melesi*. Interestingly, the dimensions of the larval suckers of *M. melesi* identified in this study were half the size of the reported dimensions of suckers in adult worms. Fixed, stained preparations of these worms were compared also with other *Mesocestoides* spp. in the collection of the Natural History Museum, London (R. A. Bray and P. Olson, personal communication) and it was concluded that *M. melesi* could not be eliminated as the identity of these worms and with the additional genetic evidence provided in this paper, it was concluded that they were most likely to be *M. melesi*. A slide with adult tapeworms has been deposited in the Natural History Museum, London, UK, under the accession number NHMUK 2019.9.23.1.

### Molecular identification of other larval and adult cestodes

Two isolates were identified as *Taenia crassiceps* based on 100% identity of the newly generated *cox*1 sequences with a sequence from the GenBank database (KY321321). One isolate was derived from an adult tapeworm from a red fox from the Mazowieckie Voivodeship and the second was a larva from the common vole, trapped in Masuria in 2000 (Table 1). Unfortunately, we were able to amplify only the *cox*1 gene fragment from the latter isolate. These two isolates grouped with other *T. crassiceps* in one clade of the phylogenetic tree based on *cox*1 sequences (Fig. 3).

The two *Hydatigera* larvae were identified as *H. kamiyai* based on 100% similarity of our *cox*1 sequences with sequences from the GenBank database (NC037071). Again, for these larval isolates from bank voles and common voles sampled in 2000, only *cox*1 and *cox*1 and *12S* rDNA sequences, respectively, were amplified successfully. These two *cox*1 sequences localized in one clade with the *H. kamiyai* reference sequences from voles [6].

We were able to obtain *cox*1 and *12S* rDNA sequences for *C. globifera* larvae from *A. agrarius.* However, we found no match with any available sequences deposited in GenBank for both markers, so the sequences were deposited as *C. globifera* based on morphological identification (number and dimensions of larval hooks).

## Discussion

In the present study, three genetic markers were used for identification of cestode species recovered from both intermediate (rodents) and definitive hosts (red fox, Eurasian lynx and Eurasian badger) with a particular focus on *Mesocestoides* spp. We demonstrated that *M. litteratus* is a dominant species, occurring in red foxes in Poland and also in the Eurasian lynx from Podkarpackie, south-east Poland and in rodents. However, four isolates from rodents from the Mazury Lake District and all three isolates from Eurasian badgers from the same region created a separate clade, distant from all known species or genotypes available in the GenBank database, but most similar to North American *M. vogae* (syn. *M. corti*) or recently described *M. canislagopodis* [16]. Although genetic divergence for *18S* rDNA between our unique isolates and these *Mesocestoides* spp. was only about 1–3%, much higher divergence was noted for the mitochondrial markers, 9–10% for *12S* rDNA and 11–12% for *cox*1, which is enough to consider that these isolates must be a distinct tapeworm species with a novel genetic signature [6, 11, 35, 36]. On balance, taking into account both our morphological observations on adult worms and the genetic analysis, the samples in this clade are most likely to represent *M. melesi*. Our larval and adult cestodes of putative *M. melesi* revealed no obvious differences with the description of *M. melesi*, a species that was first described in 1985 from the Eurasian badger *M. meles* [34]. This first robust description of *M. melesi* was based on a significant number of tapeworms from 42 Eurasian badgers from Bulgaria and detailed several morphological features enabling differentiation of these worms as a new species distinct from *M. lineatus* and *M. erschovi*. The authors did not suggest any intermediate hosts for the new species at that time.

Moreover, although our four *M. melesi* samples from rodents displayed the highest genetic similarity to *M. vogae* (syn. *M. corti*), it is unlikely that they could represent a variant of *M. vogae*. Phylogenetic analyses clearly separated our sequences from *M. vogae*. Besides, *M. corti* was described in the USA by Hoeppli [37] based on about 100 tapeworms (adults, 8 cm long) recovered from the intestines of *Mus musculus* in Colorado in 1909 and recorded in the collection of Professor W.W. Cort. Later, others found only tetrathyridia in mice and rodents and small adults in cats, dogs and skunks [38, 39]. The original description by Hoeppli [37] was eventually questioned [38], especially as the original description was based solely on one archival field sample and rodents are now known not to serve as definitive hosts of *Mesocestoides* spp. These serious concerns led to the description of a new species by Etges [39], *M. vogae*, based on metacestodes from the body cavities and livers of fence lizards (*Sceloporus occidentalis biseriatus*) from California [40]. This description was approved and *M. corti* was synonymized with *M. vogae*. However, no data on definitive hosts was presented in the description of this new species. Then in 2004, Padgett and Boyce [8] provided detailed molecular data on the definitive hosts of *M. vogae*, including coyotes (*Canis latrans*) and domestic dogs, and proposed rodents (deer mice *Peromyscus maniculatus*) as intermediate hosts of this cestode. This biological data support differentiation of *M. vogae* (syn. *M. corti*) from *M. melesi*, with its life-cycle based on Eurasian badgers and European rodents (*Myodes* spp., *Apodemus* spp.).

To the best of our knowledge, our study is one of the first presenting the molecular characteristics of tapeworms derived from both intermediate and definitive hosts. Our analyses have demonstrated clearly that larval and adult *Mesocestoides* derived from rodents and Eurasian badgers, respectively, are closely related and genetically very similar, distant from other *Mesocestoides* species/genotypes, representing a badger-specific species. Thus, taking into account the previous description of *Mesocestoides* from Eurasian badgers as a new species by Yanchev and Petrov [34], we provide evidence for recognition of *M. melesi* as a valid species.

Our study supports the dominant occurrence of *M. litteratus* in rodents and carnivores from central Europe, in accordance with previous studies [14, 15, 41]. This species appears to be a generalist, occurring in a wide range of carnivores (but not in Eurasian badgers); in our study it was found in red foxes from different regions of Poland and in a Eurasian lynx from south-east Poland (Podkarpackie Voivodeship). In a recent molecular study of tapeworms, only this *Mesocestoides* species was found in dogs and cats in south-east Poland [42]. A few years ago, tetrathyridia of *M. litteratus* were identified molecularly in *M. glareolus* and *A. agrarius* from the Wrocław area, south-west Poland [19]. Both rodent species, in which we identified *M. litteratus* larvae, *M. glareolus* and *A. flavicollis*, are known intermediate hosts of this species. Interestingly, phylogenetic analyses of *M. litteratus* mitochondrial sequences obtained in this study from carnivores and rodents revealed some degree of diversity, suggesting the existence of several genotypes within the species.

The molecular characteristics of tapeworms derived from both intermediate and final hosts allowed us to conclude that the same genotype of *T. crassiceps* was present in rodents (*M. arvalis*) and red foxes, the definitive hosts of this species.

In our previous studies, cysts containing strobilocercus larvae, morphologically identified as *T. taeniaeformis*, were found in the livers of *M. glareolus* [3–5] and *Arvicola terrestris* (Bajer, unpublished) from the same region of Poland. However, following a recent reappraisal of *H. taeniaeformis* and the description of *H. kamiyai* (previously *Taenia taeniaeformis* complex; [6, 7]), here we were able to confirm the occurrence of *H. kamiyai* in voles as intermediate hosts. Moreover, we have now added a third species of *Microtus*, the common vole *M. arvalis*, and the bank vole *Myodes glareolus* to the published list of intermediate hosts for this cestode [6]. To the best of our knowledge, the present study is also the first to report the molecular detection of *H. kamiyai* in Poland, in addition to the recent identification of *H. taeniaeformis* in cats [42].

## Conclusions

Molecular and phylogenetic analyses support the recognition of *M. melesi* as a valid species. To the best of our knowledge, our data represent the first record of the larvae of this species in rodents and the first report of the occurrence of *H. kamiyai* in rodents from Poland.

## Supplementary information

**Additional file 1: Table S1.** Similarity (%) between selected *12S* rDNA sequences of *Mesocestoides* generated in the present study and sequences of *Mesocestoides* spp. from the GenBank database. **Table S2.** Similarity (%) between selected *18S* rDNA sequences of *Mesocestoides* generated in the present study and sequences of *Mesocestoides* spp. from the GenBank database. **Table S3.** Similarity (%) between selected *cox*1 sequences of *Mesocestoides* generated in the present study and sequences of *Mesocestoides* spp. from the GenBank database.

**Additional file 2: Figure S1.** Images of the larvae of *M. melesi*. Larvae from bank vole no. 029: free larva from peritoneal cavity (**a**-c) and liver cyst (**d**).

**Additional file 3: Figure S2.** Images of the adult *M. melesi* from the Eurasian badger no. 367. **a** Scolex with suckers. **b** Uterine proglottid, cirrus pouch visible. **c** Gravid proglottids with paruterine organ and cirrus pouch. **d** Scolex and gravid proglottid.

**Additional file 4: Table S4.** Comparison of the measurements of larval and adult *M. melesi* with *M. litteratus* and data from Yanchev and Petrov [34].

## Data Availability

The datasets supporting the conclusions of this article are included within the article and its additional files. Representative sequences are submitted to the GenBank database (accession numbers are provided in Table 1). Tapeworms from three Eurasian badgers, one Eurasian lynx, and one yellow-necked mouse were deposited in the scientific collection of the MRI PAS in Białowieża, Poland. A slide with adult tapeworms *M. melesi* has been deposited in the Natural History Museum, London, UK, under the accession number NHMUK 2019.9.23.1.

## Abbreviations

PCR: polymerase chain reaction
SNP: single nucleotide polymorphism
mt: mitochondrial
