# Supplementary figures and images for: Rodents as intermediate hosts of cestode parasites of mammalian carnivores and birds of prey in Poland, with the first data on the life-cycle of Mesocestoides melesi
Source: Parasit Vectors. 2020 Feb 22;13:95. doi: 10.1186/s13071-020-3961-2 (PMC7036256; doi:10.1186/s13071-020-3961-2)

## Slide 1
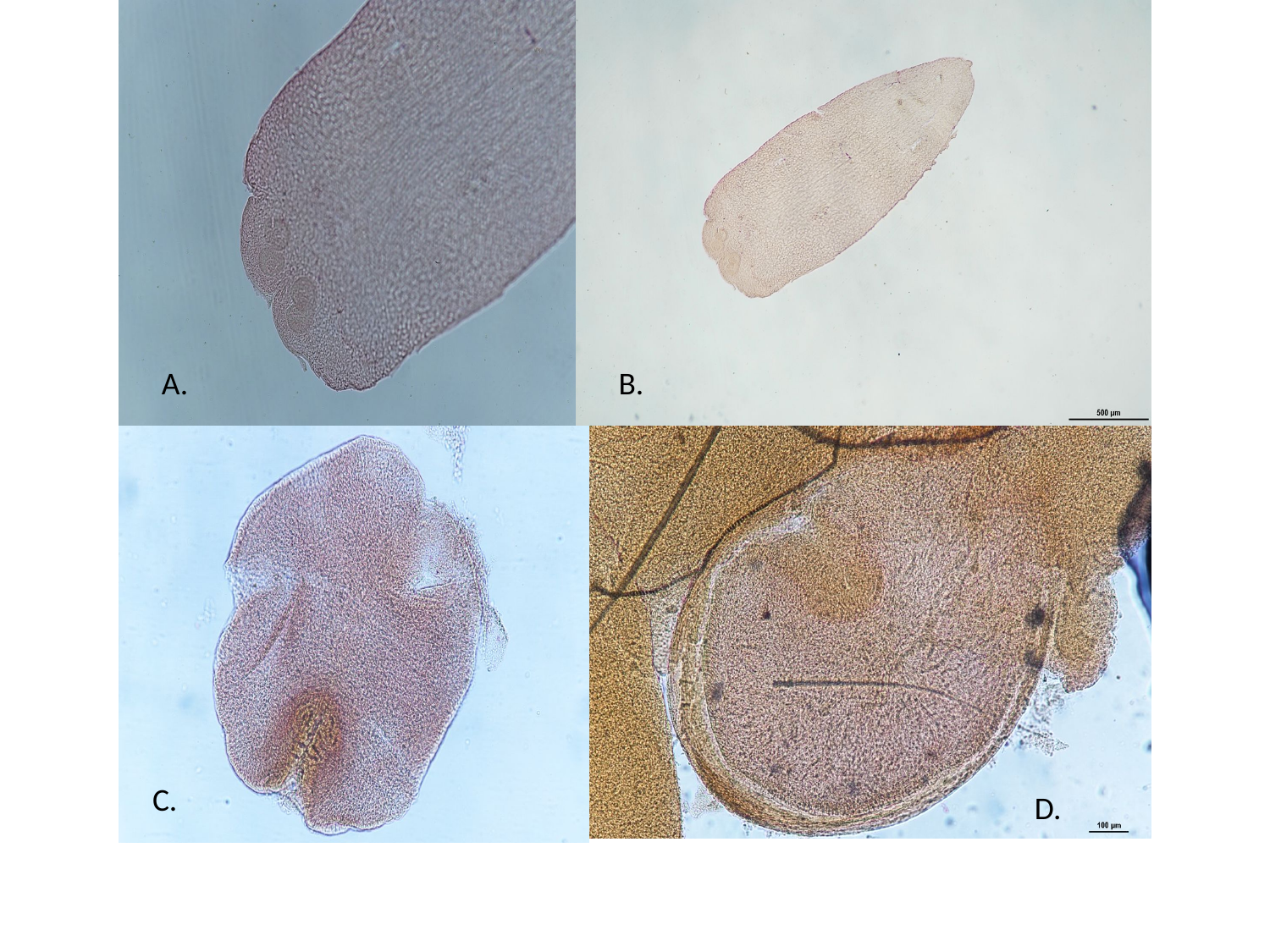

A.
B.
C.
D.

Supplement: Supplementary file 2 — Additional file 2: Figure S1. Images of the larvae of M. melesi. Larvae from bank vole no. 029: free larva from peritoneal cavity (a-c) and liver cyst (d). [file 13071_2020_3961_MOESM2_ESM.pptx]

## Slide 1
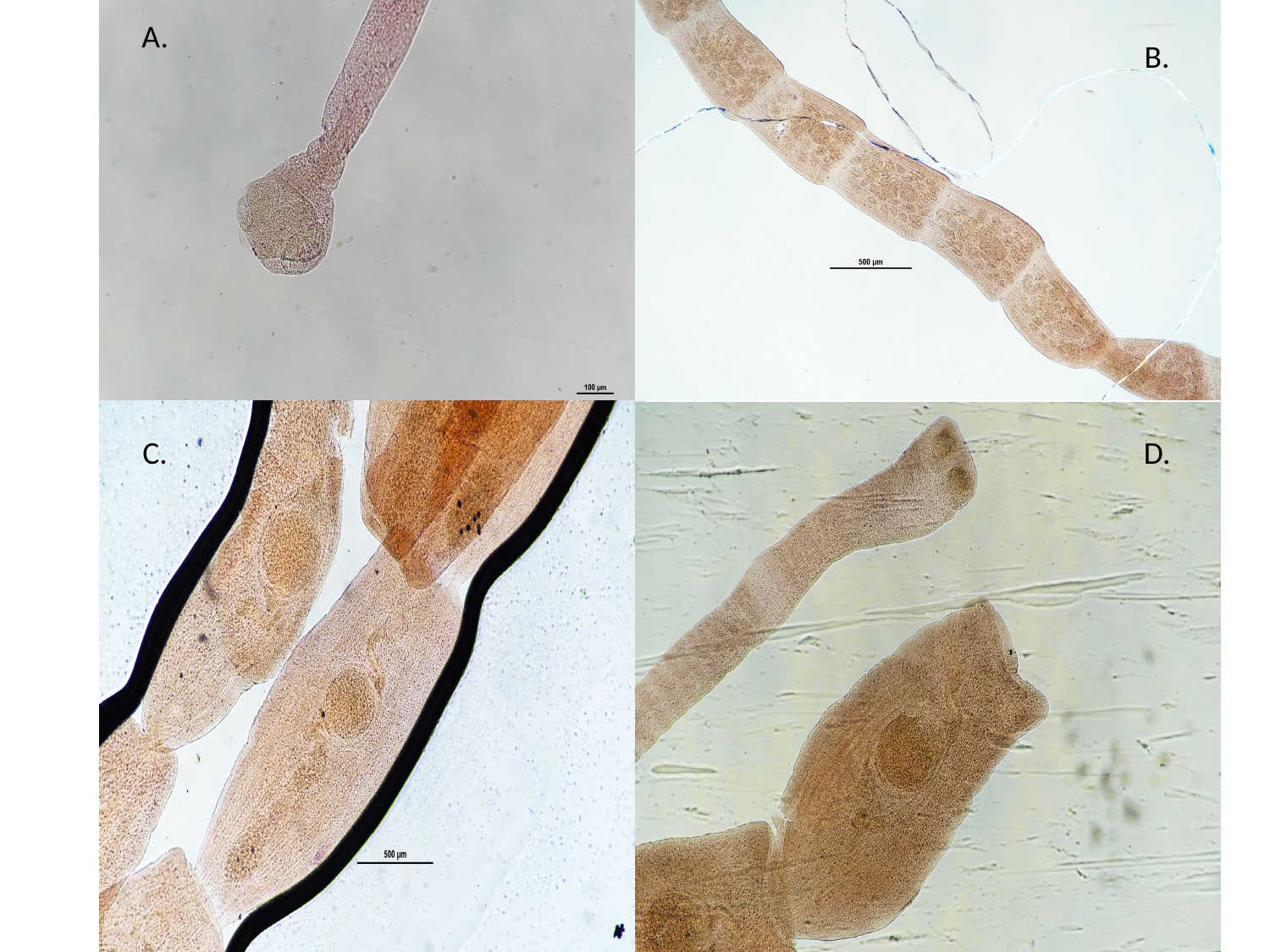

A.
B.
C.
D.

Supplement: Supplementary file 3 — Additional file 3: Figure S2. Images of the adult M. melesi from the Eurasian badger no. 367. a Scolex with suckers. b Uterine proglottid, cirrus pouch visible. c Gravid proglottids with paruterine organ and cirrus pouch. d Scolex and gravid proglottid. [file 13071_2020_3961_MOESM3_ESM.pptx]
