# Supplementary material for: Rodents as intermediate hosts of cestode parasites of mammalian carnivores and birds of prey in Poland, with the first data on the life-cycle of Mesocestoides melesi
Source: Parasit Vectors. 2020 Feb 22;13:95. doi: 10.1186/s13071-020-3961-2 (PMC7036256; doi:10.1186/s13071-020-3961-2)
Supplement: Supplementary file 4 — Additional file 4: Table S4. Comparison of the measurements of larval and adult M. melesi with M. litteratus and data from Yanchev and Petrov [34]. [file 13071_2020_3961_MOESM4_ESM.docx]

Additional file 4: Table S4. Comparison of selected measurements of larval and adult *Mesocestoides litteratus* and *Mesocestoides melesi,* including reference values from the description by Yanchev & Petrov [33].

| Object | n | Mean (µm) | Range | |
| --- | --- | --- | --- | --- |
| *M. litteratus* larvae (no. 130) |  |  | Min | Max |
| - body length | 5 | 2175 | 2047 | 2474 |
| - body width | 5 | 1833 | 1790 | 2133 |
| - sucker length | 12 | 202 | 144 | 253 |
| - sucker width | 12 | 166 | 140 | 188 |
| *M. melesi* larvae |  |  |  |  |
| - body length | 10 | 1297 | 865 | 1895 |
| - body width | 10 | 972 | 724 | 1261 |
| - sucker length | 10 | 84 | 77 | 94 |
| - sucker width | 10 | 71 | 61 | 83 |
| *M. melesi* adult |  |  |  |  |
| - sucker lenght | 4 | 167 | 151 | 186 |
| -sucker width | 4 | 152 | 137 | 165 |
| Yanchev and Petrov (1985): |  |  |  |  |
| *M. melesi* adult |  |  |  |  |
| - sucker lenght | 72 | 148 | 109 | 188 |
| -sucker width | 72 | 130 | 93 | 168 |
